# Individual exercise training response and ECG morphology in patients after an acute coronary syndrome

**DOI:** 10.14814/phy2.70687

**Published:** 2025-12-03

**Authors:** Elina Roiha, Juha Perkiömäki, Antti M. Kiviniemi, Juhani Junttila, Heikki V. Huikuri, Mikko P. Tulppo

**Affiliations:** ^1^ Research Unit of Biomedicine and Internal Medicine University of Oulu Oulu Finland; ^2^ Medical Research Center Oulu Oulu University Hospital and University of Oulu Oulu Finland

**Keywords:** cardiac patients, electrocardiography, exercise capacity, heart rate variability

## Abstract

Patients after an acute coronary syndrome (ACS) with preserved left ventricle ejection fraction (LVEF) (*n* = 45, 10 female) were guided by exercise training for 6 months after percutaneous coronary intervention. Exercise capacity, LVEF, standard resting ECG, and 24‐h Holter recording were measured at baseline. Time and frequency domain heart rate variability were analyzed over 24 h. Sokolow–Lyon index, as a marker of left ventricular hypertrophy and P wave Terminal Force in lead V_1_ (PTF), a marker of left atrial dilatation, along with the standard parameters were analyzed from ECG. A training load of over 6 months (TRIMP) was calculated. Subjects were categorized according to training responses in exercise capacity as low (−3 ± 4%), average (6 ± 2%), and high (15 ± 6%) responders. PTF and P wave duration at baseline differed between the groups: 0.051 ± 0.018, 0.028 ± 0.023, and 0.016 ± 0.015 ms (*p* < 0.0001) and 120 ± 11, 113 ± 12 and 104 ± 13 ms (*p* = 0.002) for low, average, and high groups, respectively. Other ECG variables, or any clinical parameters (e.g., sex, age, baseline fitness, LVEF) at baseline or TRIMP were not associated with the training response. Left atrial dilatation, as indicated by PTF on surface ECG, is associated with a low training response in exercise capacity in patients after ACS.

## 1 INTRODUCTION

Exercise training and physical activity are core components of secondary prevention in coronary artery disease (Balady et al., 2007). Exercise‐based cardiac rehabilitation reduces both cardiac and all‐cause mortality (Taylor et al., 2004). Current guidelines recommend at least 150 minutes of low‐to‐moderate intensity aerobic exercise per week, along with two resistance training sessions, for secondary prevention in these patients (Fihn et al., 2012). However, significant individual differences in exercise training responses have been observed in both healthy individuals (Bouchard & Rankinen, 2001; Hautala et al., 2003) and cardiac patients (Karjalainen et al., 2015) typically measured by maximal oxygen uptake, maximal workload, or metabolic equivalents (METs). The physiological background or mechanisms underlying individual training responses in patients with coronary artery disease remain unknown.

Exercise training response, assessed by change in aerobic exercise capacity, has been associated with sex, age, baseline fitness (Bouchard & Rankinen, 2001) and baseline heart rate variability (HRV) in healthy subjects (Hautala et al., 2003) and obese individuals with or without type 2 diabetes (Baynard et al., 2014). Demographic factors account for approximately 10%–15% of individual variability in exercise training responses among healthy participants (Bouchard & Rankinen, 2001). Cardiac autonomic function alone, measured by vagally mediated beat‐to‐beat HRV over 24‐h recordings, explains up to 20% of this variability in middle‐aged, healthy subjects (Hautala et al., 2003). Individuals with high levels of cardiac vagal modulation prior to exercise training, measured by HRV, tend to achieve greater improvements in exercise capacity compared to those with lower vagal modulation, despite following the same training program. However, the association between HRV and exercise training response in patients with coronary artery disease remains unknown.

Cardiac structure and function are closely associated with exercise capacity in cardiac patients, as stroke volume and cardiac output are the primary limiting factors of exercise capacity (Lundby et al., 2017). Several cross‐sectional studies have demonstrated that left atrial structure and function are independently associated with exercise capacity in cardiac patients with preserved ejection fraction (Kusunose et al., 2012; Maffeis et al., 2021), patients with type 2 diabetes (Vukomanovic et al., 2020), and individuals without any structural heart disease (Leite et al., 2017). It is well established that dilatation of atria (Hazen et al., 1991; Miller et al., 1983) and ventricles (Sokolow & Lyon, 1949) leads to significant changes in traditional 12‐lead electrocardiography (ECG) morphology. Therefore, ECG morphology assessed prior to structured exercise training programs may provide new insights into the mechanisms underlying individual training responses in cardiac patients.

Morris et al. (1964) first introduced the P‐wave Terminal Force in lead V_1_ (PTF) by multiplying the duration (in seconds) and the amplitude (in millimeters) of the negative terminal deflection of the P wave. The authors demonstrated that patients with PTF ≥0.04 ms had a higher incidence of left‐sided valvular lesions, and that abnormal PTF also reflected disease severity (Morris et al., 1964). More recently, Eranti et al. (2014) reported that individuals from a middle‐aged general population with PTF ≥0.06 ms had an increased risk of atrial fibrillation and all‐cause mortality over a 35‐year follow‐up period (Eranti et al., 2014). Most importantly, abnormal PTF is particularly associated with left atrial dilatation in cardiac patients (Hazen et al., 1991; Miller et al., 1983). Additionally, an abnormal PTF on ECG is a well‐known marker of impaired left ventricle diastolic function in patients with preserved LVEF (Tanoue et al., 2017).

This study addressed whether baseline HRV and ECG variables, in addition to clinical characteristics, are associated with individual exercise training responses among ACS patients. Additionally, we assessed any changes in exercise capacity, HRV, and ECG variables among ACS patients, who underwent exercise training intervention. The patients underwent a controlled, 6‐month, home‐based exercise training program guided by current cardiac rehabilitation guidelines.

## 2 METHODS

### 2.1 Study population

The study patients were recruited from a consecutive series of patients admitted to Oulu University Hospital due to ACS from December 2017 to January 2019 (ClinicalTrials.gov identifier: NCT03704025). Left ventricle ejection fraction (LVEF) was measured before the exercise training program in the hospital. An experienced cardiologist performed a transthoracic 2‐dimensional echocardiography using a General Electric Vivid E9 device with an M5S‐D 1.5/4.6 MHz sector transducer for cardiovascular imaging (GE Health Medical, Horten, Norway). The measurements were performed according to the guidelines of the American Society of Echocardiography (Lang et al., 2006). All patients underwent coronary angiography and were treated by PCI (769 screened patients). A cardiologist assessed each patient's eligibility based on clinical status and the following exclusion criteria: age under 18 or over 70; left ventricle heart failure; Canadian Cardiac Society (CCS) class angina pectoris symptoms ≥2; implanted or planned cardioverter defibrillator or pacemaker; chronic atrial fibrillation; participation in a competing clinical trial; severe peripheral atherosclerosis, retinopathy or neuropathy; dementia; life expectancy <2 years due to other serious diseases. A total of 618 patients were excluded. The most common reasons for exclusion were age over 70 years (*n* = 149), left ventricular heart failure (*n* = 151), and other reasons (*n* = 247), such as an implanted or planned cardioverter‐defibrillator, planned coronary artery bypass surgery, or severe comorbidities (e.g., cancer or unable to participate in exercise training due to musculoskeletal problems). Although 151 patients met the enrollment criteria, 101 declined to participate in the study. Ultimately, 50 patients were enrolled in the exercise training protocol.

To control the exercise training intervention for the ACS patients' group, we required age‐ and sex‐matched healthy control subjects via an ad in a local newspaper. Control subjects' inclusion criteria were aged 50–70, nonsmoking, and a body mass index (BMI) of <30 kg/m^2^. Exclusion criteria included any cardiovascular or pulmonary diseases, as well as the use of any medication. The participants were informed about the study (description, subject information, and consent document). The study was performed according to the Declaration of Helsinki; the local committee of research ethics of the Northern Ostrobothnia Hospital District approved the protocol, and all the subjects provided written informed consent. The flow chart of the patients' requirements was published recently (Saarikoski et al., 2024).

### 2.2 Study design and methods

Patients were randomized into a virtual autonomous physiotherapy agent group (VAPA) (*n* = 25) and a traditionally guided exercise training group (*n* = 25) before baseline measurements (Saarikoski et al., 2024). However, there were no differences in exercise capacity responses between the patient groups; therefore, they were pooled in the present study. Three subjects discontinued the exercise training (due to lack of motivation, car accident, and a new severe noncardiac disease), 24‐h ECG recording was corrupted for two subjects. Thus, the final number of patients included in the analysis was 45. Healthy control subjects were instructed to continue their normal daily activities over a 6‐month period. All study participants underwent a comprehensive clinical examination, standard 12‐lead ECG recordings at rest in the supine position, a cycle ergometer exercise test, and had 24‐h ambulatory ECG recordings at baseline and 6 months after the intervention. All the baseline measurements for patients were conducted 1–3 weeks after hospital discharge following PCI. According to current guidelines on myocardial revascularization, exercise testing for functional evaluation and exercise training prescription can be safely performed 7–14 days after PCI (Task Force on Myocardial Revascularization of the European Society of Cardiology (ESC) and the European Association for Cardio‐Thoracic Surgery (EACTS), European Association for Percutaneous Cardiovascular Interventions (EAPCI), et al., 2010). However, in this study, baseline exercise testing was postponed to 3 weeks after PCI due to logistical reasons.

### 2.3 Exercise training

The exercise training intervention lasted 6 months. According to current guidelines (European Association of Cardiovascular Prevention and Rehabilitation Committee for Science Guidelines, EACPR, et al., 2010; Fletcher et al., 2013) patients were prescribed 4–5 aerobic sessions and two strength training sessions per week. All patients received individual guidance using the Rating of Perceived Exertion (RPE) scale () to assess the average intensity of each exercise session (Borg, 1982). Patients recorded the exercise mode, duration, and mean RPE of each session in a diary or on a tablet computer.

Aerobic training consisted of walking or cycling at the intensity corresponding to an RPE of 10–15. Strength training, primarily targeting the lower limbs, was performed at home and at a low to moderate intensity (RPE of 10–15). Training impulse (TRIMP) was calculated from the exercise diaries using a computer, by multiplying the RPE by the duration of each session, as previously described (Foster, 1998; Hautala et al., 2017).

### 2.4 Exercise tests and resting ECG

Exercise capacity (maximal load, Watts) was measured using a cycle ergometer test, starting from 30 Watts and increasing by 10 Watts per minute for women and 15 Watts per minute for men (Karjalainen et al., 2015) until voluntary exhaustion (Monark 839E, Sweden). ECG was continuously monitored and recorded throughout the test, and blood pressure was measured at every second load. A standard resting ECG was recorded after a 10‐minute rest in the supine position and analyzed using CardioSoft version 6.5 (GE Healthcare, Fairfield, CT).

The Sokolow–Lyon index, as a measure of ventricle hypertrophy, along with standard ECG parameters including P wave duration, PR interval, QRS interval, QT interval, and corrected QT interval (Bazett's formula; QTc) was automatically analyzed from 12‐lead ECG recordings taken before and after the intervention.

**FIGURE 1:**
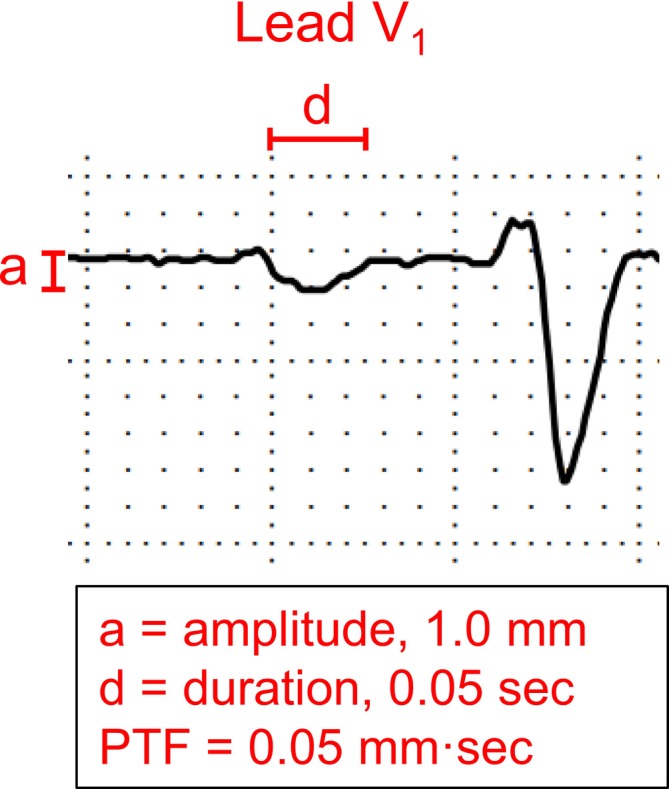
An example of P wave terminal force calculation for one subject from lead V_1_ (A).

PTF, indicator of left atrial dilatation, was calculated as previously described and analyzed as a continuous variable (Figure 1) (Hazen et al., 1991; Miller et al., 1983). PTF was also categorized into two groups: 0.00–0.04 ms and >0.04 ms. A PTF value >0.04 ms has been associated with an increased risk of cardiac death and congestive heart failure (Eranti et al., 2014). PTF was classified as present if the negative deflection amplitude exceeded 0.25 mm and the duration was greater than 20 ms, and therefore the PTF was classified as absent if either the amplitude or duration were less than the accuracy values. Amplitude was measured with an accuracy of 0.25 mm and a duration with an accuracy of 10 ms. The individual who analyzed PTF (M.P.T.) was blinded to group assignment and to all phenotypic information, including age, sex, and exercise training response. A second investigator (A.M.K.) extracted and magnified lead V_1_ ECG traces from the original PDF files, ensuring removal of all identifying information. Subsequently, M.P.T. performed PTF measurements on the zoomed lead V_1_ ECG images. Finally, A.M.K. appended the PTF data to the original dataset. Automatic digital analysis was not available. Therefore, PTF measurements were performed manually through visual inspection.

### 2.5 Heart rate variability

Ambulatory 24‐h ECG was obtained using the eMotion Faros 360° device (Bittium Corporation, Oulu, Finland; sampling frequency: 1000 Hz) to analyze 24‐h HRV. All R‐R intervals were visually inspected using ECG segments to identify and exclude technical artifacts and ectopic beats, which accounted for less than 2% of beats in each subject. Artifacts and ectopic beats were corrected using the local average method. However, sequences containing ≥10 consecutive noisy or ectopic beats were excluded from analysis.

R‐R interval dynamics were calculated from the entire 24‐h recording period. Recordings were conducted on nonexercise days for both groups. At the end of the training intervention, recordings were performed after a 48‐h nonexercise period (Tulppo et al., 2003). The mean R‐R interval was calculated, and the standard deviation of all R‐R intervals (SDNN) was used as a time‐domain measure of HRV.

Power spectral density of R‐R interval variability was estimated using an autoregressive model (order 20). Very low‐frequency (VLF) power (0.0033–0.04 Hz) was calculated from the full 24‐h segment, while low‐frequency (LF) power (0.04–0.15 Hz) and high‐frequency (HF) power (0.15–0.4 Hz) were calculated from segments of 512 R‐R intervals sampled throughout the 24‐h recording (Heart rate variability, 1996). Spectral values are presented as absolute values after logarithmic transformation. The LF/HF ratio was also calculated as a marker of sympathovagal balance (Pagani et al., 1997).

### 2.6 Statistics

The normality of variable distributions was assessed using the Kolmogorov–Smirnov goodness‐of‐fit test. If a variable was not normally distributed, a logarithmic transformation was applied prior to statistical analysis, or nonparametric methods appropriate for nonnormal distributions were used. Analysis of variance for repeated measurements (ANOVA) was employed to assess the significance of the intervention and to compare groups over time (main effects: time, group, and time × group interaction). When a significant time × group interaction was detected, an appropriate post hoc analysis was conducted. Chi‐square test or Mann–Whitney *U* tests were used to compare categorical and nonparametric variables, respectively, as appropriate.

Patients were divided into tertiles based on the change in maximal workload (Watts) to examine the association between training response and baseline characteristics (e.g., lipid profile, glycemic control, medical history, HRV, and ECG variables). One‐way analysis of variance (ANOVA) was used to compare baseline characteristics between these tertiles (Tables 3 and 4). Bonferroni's post hoc tests were applied when appropriate.

Analysis of covariance (ANCOVA) was conducted to examine the interaction between sex and PTF (as a categorical variable) in relation to the exercise training response in exercise capacity. Additionally, stepwise linear regression was used to assess the association between exercise training response (dependent variable) and potential predictors, including age, sex, BMI, baseline exercise capacity, type 2 diabetes, and baseline PTF (as a continuous variable).

## 3 RESULTS

### 3.1 Baseline characteristics

Table 1 shows the baseline characteristics of patients and healthy controls. There were no differences in sex or age between patients (age range 47–70 years) and healthy controls (age range 50–70 years). Compared with healthy controls, patients with ACS had higher body weight and BMI, and exhibited poorer glycemic control, evidenced by elevated glycated hemoglobin levels.

**TABLE 1:** Characteristics of patients after acute coronary syndrome and healthy controls at baseline.

Variable	Controls *n* = 27	Patients *n* = 45	*p*‐Value
Men, %	17 (63)	35 (76)	0.192
Age, years	62 ± 5	62 ± 8	0.940
Height, cm	170 ± 9	171 ± 6	0.551
Weight, kg	73 ± 13	85 ± 12	<0.001
Body mass index, kg/m^2^	25 ± 3	29 ± 5	<0.001
Systolic BP, mmHg	137 ± 15	134 ± 18	0.341
Diastolic BP, mmHg	86 ± 8	84 ± 11	0.293
Glycated hemoglobin (mmol/mol)	33 ± 3	41 ± 10	<0.001
Total cholesterol (mmol/L)	—	4.16 ± 1.30	
HDL cholesterol (mmol/L)	—	1.16 ± 0.26	
LDL cholesterol (mmol/L)	—	2.26 ± 1.13	
Triglycerides (mmol/L)	—	1.65 ± 0.95	
Current smokers (%)	0	3 (6)	
LVEF, %	—	63 ± 9	
**Medical history**			
History of acute MI (%)	0	6 (13)	
History of revascularization (%)	0	12 (27)	
Diabetes mellitus (%)	0	6 (13)	
**Medication**			
β‐blockers (%)	0	27 (60)	
ACE or ATII blockers (%)	0	39 (87)	
Calcium channel blockers (%)	0	15 (33)	
Diuretics (%)	0	5 (11)	
Anticholesterol agents (%)	0	45 (100)	
**Training**			
Aerobic training, total TRIMP × 1000	—	51 ± 29	
Resistance training, total TRIMP × 1000	—	10 ± 7	
Total training, times/6 months	—	114 ± 32	
Total training, total TRIMP × 1000	—	61 ± 32	

*Note*: Values are mean ± standard deviation, *n* (% within group).

Abbreviations: ACE, angiotensin‐converting enzyme; ATII, angiotensin receptor II; BP, blood pressure; HDL, high‐density lipoprotein; LDL, low‐density lipoprotein; LVEF, left ventricle ejection fraction; MI, myocardial infarction; TRIMP; training impulse.

### 3.2 Effects of exercise training

Table 2 shows exercise capacity, HRV, and resting ECG variables before and after the intervention for patients and controls. When all time points were considered, measures of exercise capacity and the LF/HF ratio were lower, and P wave duration was longer in patients than in healthy controls. PTF was higher in patients as a continuous variable, and the prevalence of PTF >0.04 ms was more common in patients, when all timepoints were considered (11% vs. 15% in controls, and 38% vs. 38% in patients at baseline and after intervention, respectively, *p* = 0.014). There was a greater postintervention increase in maximal exercise load in ACS patients than in controls according to the time × group interaction. HRV indices or ECG variables did not change after intervention.

**TABLE 2:** Exercise capacity, heart rate variability over 24 h, and 12‐lead ECG parameters in patients after acute coronary syndrome and healthy control subjects at baseline and after intervention.

Variable	Controls *n* = 27		Patients *n* = 45		*p*‐Value ANOVA for repeated measures		
	Pre	Post	Pre	Post	Time	Group	Interaction
**Exercise capacity**							
METs	9.2 ± 2.2	9.4 ± 2.2	7.1 ± 1.9	7.6 ± 2.0	<0.001	<0.001	0.180
Max load, W	174 ± 50	178 ± 52	149 ± 40	160 ± 43	<0.001	0.047	0.043
Max heart rate, bpm	162 ± 12	162 ± 13	140 ± 17	142 ± 18	0.491	<0.001	0.187
**24 h recording**							
Heart rate, bpm	71 ± 9	69 ± 8	68 ± 7	68 ± 8	0.402	0.345	0.359
SDNN, ms	148 ± 31	151 ± 28	150 ± 31	153 ± 37	0.387	0.797	0.970
VLF power, ln ms^2^	7.16 ± 0.60	7.19 ± 0.50	7.28 ± 0.48	7.31 ± 0.51	0.552	0.355	0.943
LF power, ln ms^2^	6.45 ± 0.73	6.39 ± 0.67	6.31 ± 0.68	6.35 ± 0.73	0.874	0.616	0.263
HF power, ln ms^2^	5.19 ± 0.90	5.23 ± 0.77	5.40 ± 0.72	5.50 ± 0.83	0.266	0.229	0.605
LF/HF ratio	3.99 ± 2.29	3.60 ± 1.99	2.94 ± 1.50	2.89 ± 1.71	0.231	0.044	0.366
**12‐lead ECG**							
RR interval, ms	999 ± 165	985 ± 165	965 ± 147	965 ± 143	0.562	0.452	0.593
P wave duration, ms	106 ± 13	103 ± 15	112 ± 13	111 ± 16	0.383	0.020	0.717
PTF, ms	0.016 ± 0.015	0.015 ± 0.017	0.032 ± 0.023	0.036 ± 0.030	0.390	<0.001	0.277
PTF >0.04, *n* (%)	3 (11)	4 (15)	17 (38)	17 (38)	0.672	0.014	0.672
PR‐interval, ms	168 ± 43	162 ± 28	170 ± 24	171 ± 24	0.216	0.450	0.098
QRS‐interval, ms	92 ± 9	91 ± 9	100 ± 21	100 ± 21	0.389	0.054	0.348
QT, ms	410 ± 26	411 ± 26	415 ± 30	415 ± 31	0.740	0.515	0.976
QTc	412 ± 19	415 ± 19	423 ± 25	424 ± 23	0.331	0.063	0.454
Sokolow–Lyon, mm	23.8 ± 6.1	22.6 ± 6.2	20.4 ± 5.8	20.2 ± 6.6	0.011	0.054	0.070

*Note*: Values are mean ± standard deviation, *n*, number of patients PTF >0.04 within group (% within group). Time is a comparison of pre‐ and postintervention measures within all study subjects combined, and that Group is a comparison between control and patient cohorts within all timepoints combined.

Abbreviations: HF, high‐frequency power (0.015–0.4 Hz); LF, low‐frequency power (0.04–0.15 Hz); METs, metabolic equivalents; PTF, P wave terminal force; SDNN, standard deviation of all R‐R intervals; VLF, very low‐frequency power (0.0033–0.04 Hz).

### 3.3 Determinants of individual training response in exercise capacity among ACS patients

Table 3 shows the baseline characteristics of the ACS patients divided according to their exercise training responses. Baseline exercise capacity was not associated with training response in any group (Table 3). The change in Watts from baseline to the end of the training was −3.0% ± 4.3%, +6.2% ± 2.2%, and + 14.8% ± 5.5% for low, average, and high responders, respectively (*p* < 0.001). After Bonferroni correction, there were no differences in any clinical parameters or training variables among the low, average, and high responders (Table 3).

**TABLE 3:** Characteristics of patients after acute coronary syndrome before exercise intervention according to exercise training response in Watts. Patients are categorized according to training responses in exercise capacity as low, average, and high responders.

Variable	Low *n* = 15	Average *n* = 15	High *n* = 15	ANOVA *p*‐value
Baseline exercise capacity, W	151 ± 48	151 ± 26	143 ± 43	0.818
Δ Exercise capacity, W %	−3.0 ± 4.3	6.2 ± 2.2	14.8 ± 5.5	<0.001
Baseline exercise capacity, METs	7.3 ± 2.3	7.4 ± 1.1	6.7 ± 2.0	0.527
Δ Exercise capacity, METs, %	−3.3 ± 5.2	5.8 ± 2.2	13.5 ± 4.4	<0.001
Men, %	10 (67)	12 (80)	12 (80)	0.618
Age, years	64 ± 9	62 ± 7	61 ± 10	0.675
Body mass index, kg/m^2^	29 ± 5	28 ± 4	30 ± 5	0.371
Systolic BP, mmHg	137 ± 21	130 ± 16	134 ± 17	0.511
Diastolic BP, mmHg	84 ± 13	83 ± 10	82 ± 10	0.880
HbA1C, mmol/mol	45 ± 14	38 ± 4	38 ± 3	0.087
Total cholesterol, mmol/L	4.2 ± 1.5	4.4 ± 1.1	3.8 ± 1.3	0.457
HDL cholesterol, mmol/L	1.1 ± 0.3	1.2 ± 0.3	1.2 ± 0.2	0.789
LDL cholesterol, mmol/L	2.3 ± 1.2	2.5 ± 1.0	2.0 ± 1.2	0.445
Triglycerides, mmol/L	1.9 ± 1.1	1.8 ± 1.1	1.3 ± 0.6	0.378
Current smokers (%)	1 (7)	2 (13)	0 (0)	0.343
LVEF, %	60 ± 11	65 ± 6	65 ± 8	0.179
**Exercise capacity**				
Maximal load, Watts	152 ± 48	151 ± 26	143 ± 43	0.818
METs	7.3 ± 2.3	7.4 ± 1.1	6.7 ± 2.0	0.527
Maximal heart rate, bpm	142 ± 15	142 ± 18	137 ± 19	0.711
**Medical history**				
History of acute MI (%)	3 (20)	1 (7)	2 (13)	0.562
History of revascularization (%)	6 (40)	3 (20)	3 (20)	0.360
Diabetes mellitus (%)	3 (20)	1 (7)	2 (13)	0.562
**Medication**				
β‐blockers (%)	11 (73)	9 (60)	7 (47)	0.329
ACE or ATII blockers (%)	14 (93)	10 (67)	15 (100)	0.018^a^
Calcium channel blockers (%)	2 (13)	9 (60)	4 (27)	0.020^a^
Diuretics (%)	1 (7)	2 (13)	2 (7)	0.799
Anticholesterol agents (%)	15 (100)	15 (100)	15 (100)	—
**Training**				
Aerobic training, total TRIMP × 1000	40 ± 17	60 ± 40	53 ± 23	0.134
Resistance training, total TRIMP × 1000	8 ± 7	14 ± 7	9 ± 6	0.041^a^
Total training, sessions/6 months	103 ± 35	123 ± 31	116 ± 29	0.224
Total training, total TRIMP × 1000	48 ± 20	74 ± 42	63 ± 26	0.072

*Note*: Values are mean ± standard deviation, *n* (% within group).

Abbreviations: ACE, angiotensin‐converting enzyme; ATII, angiotensin receptor II; BP, blood pressure; HDL, high‐density lipoprotein; LDL, low‐density lipoprotein; LVEF, left ventricular ejection fraction.

^a^ No significant differences exist between groups after the posthoc test with Bonferroni correction.

**FIGURE 2:**
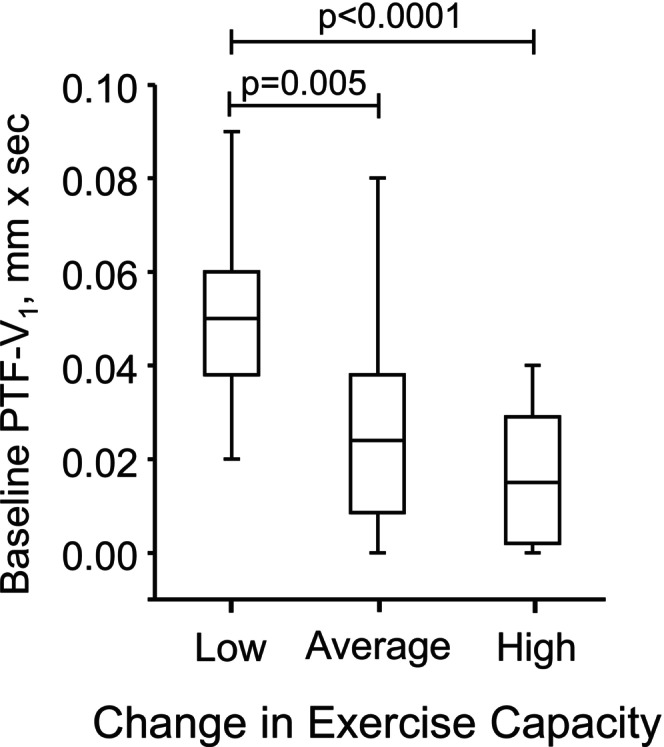
Box‐and‐whisker plot showing: The median (line in box), interquartile range (box), and range (whiskers) of baseline P wave terminal force across different exercise response groups (B). Patients were divided into tertiles based on the change in exercise capacity, categorized as low, average and high responders.

Table 4 shows baseline HRV and ECG variables for low, average, and high responders. Only P wave duration and PTF differed between groups. P wave duration differed only between the low and high responder groups, whereas PTF significantly distinguished between low vs. average and low versus high responder groups (Figure 2). Sex did not modify the association between baseline PTF and exercise training response (sex × PTF interaction *p* = 0.081). In stepwise linear regression analysis, exercise training response was used as the dependent variable, and age, sex, BMI, baseline exercise capacity, type 2 diabetes, and baseline PTF were included as predictor variables. Baseline PTF was the only variable that remained in the model (*R* = 0.46, unstandardized *β* = −167, 95% confidence interval from −265 to −70, *p* = 0.001).

**TABLE 4:** Heart rate variability over 24 h and 12‐lead ECG parameters before exercise intervention in patients after acute coronary syndrome. Patients are categorized according to training responses in exercise capacity as low, average, and high responders.

Variable	Low *n* = 15	Average *n* = 15	High *n* = 15	ANOVA *p*‐value
**24‐h recording**				
Heart rate, bpm	66 ± 8	70 ± 7	69 ± 5	0.327
SDNN, ms	159 ± 33	143 ± 28	149 ± 32	0.402
VLF power, ln ms^2^	7.44 ± 0.48	7.35 ± 0.48	7.41 ± 0.58	0.965
LF power, ln ms^2^	6.43 ± 0.45	6.17 ± 0.70	6.37 ± 0.88	0.867
HF power, ln ms^2^	5.58 ± 0.83	5.10 ± 0.62	5.50 ± 0.63	0.418
LF/HF ratio	2.89 ± 1.50	3.32 ± 1.65	2.83 ± 1.56	0.653
**12‐lead ECG recording**				
RR interval, ms	957 ± 186	958 ± 90	981 ± 160	0.880
P wave duration, ms	120 ± 11	113 ± 12	104 ± 13^b^	0.002
PTF, ms	0.051 ± 0.018	0.028 ± 0.023^a^	0.016 ± 0.015^c^	<0.001
PTF >0.04, *n* (%)	11 (73)	4 (27)^a^	2 (13)^c^	0.002
PR‐interval, ms	173 ± 26	170 ± 16	168 ± 31	0.851
QRS‐interval, ms	99 ± 21	104 ± 24	96 ± 18	0.572
QT, ms	415 ± 37	412 ± 23	417 ± 31	0.919
QTc	426 ± 23	421 ± 25	423 ± 27	0.840
Sokolow–Lyon index, mm	22 ± 6	22 ± 6	20 ± 4	0.503

*Note*: Values are mean ± standard deviation, *n*, number of patients PTF >0.04 within group (% within group).

Abbreviations: HF, high‐frequency power (0.15–0.4 Hz); LF, low‐frequency power (0.04–0.15 Hz); SDNN, standard deviation of all R‐R intervals; VLF, very low‐frequency power (0.0033–0.04 Hz).

^a^ *p* < 0.05 between average and low groups.

^b^ *p* < 0.01.

^c^ *p* < 0.001 between low and high groups, according to the posthoc test after the Bonferroni correction.

## 4 DISCUSSION

The main finding of the present study is that baseline PTF was strongly associated with individual training response in patients after ACS. Patients with a low training response had higher absolute PTF values and a higher prevalence of PTF (>0.04 ms) before the training period compared to those with average or high training responses. Second, the baseline 24‐h HRV was not associated with subsequent individual training responses in patients after ACS. Finally, the training followed current guidelines, and both resistance and aerobic exercise were carefully monitored using a daily diary. Training was quantified based on the number of training sessions, exercise intensity, and TRIMP. However, the number of training sessions, TRIMP, and baseline clinical parameters were not associated with training response.

### 4.1 P wave terminal force and training response

Although the patients in the present study had no symptoms of heart failure, they may have had mild systolic or particularly diastolic dysfunction, as indicated by the ECG sign of PTF. This may explain why these patients did not benefit from exercise training. It remains to be determined whether patients with cardiac dysfunction are truly unable to improve their exercise capacity through training programs.

Modern strain analysis using tissue Doppler and echocardiography techniques has been used to evaluate left atrial volume and, particularly, left atrial function. Strain analysis of left atrial function is measured throughout the cardiac cycle, enabling evaluation of the left atrial reservoir, conduit, and contractile functions (Elliott et al., 2023; Gan et al., 2018). Several cross‐sectional studies have highlighted the independent association between left atrial function and reduced exercise capacity in healthy subjects without structural heart disease (Leite et al., 2017), cardiac patients with preserved ejection fraction (Kusunose et al., 2012; Maffeis et al., 2021) and patients with type 2 diabetes and normal left ventricle function (Vukomanovic et al., 2020).

### 4.2 P wave duration and training response

P wave duration was also associated with the training response. The P wave duration is a marker of atrial depolarization, measured from the beginning to the end of the P wave; a normal P wave duration is less than 120 ms (Meek & Morris, 2002). A prolonged P wave duration in the present study is likely related to delayed left atrial activation, resulting in high PTF values due to left atrial dilatation. Furthermore, we can speculate that cardiac fibrosis of the left atrium may explain the prolonged P wave duration (Tiffany Win et al., 2015) and could contribute to a low exercise training response. Maximal exercise capacity is primarily regulated by the maximal capacity for oxygen delivery, which largely depends on cardiac output (Lundby et al., 2017). A dilatated left atrium, along with potential diastolic dysfunction of the left ventricle, may impair cardiac output and thereby limit exercise capacity.

### 4.3 Clinical characteristics and training response

After ACS, our patients began a highly controlled 6‐month home exercise training program 1–3 weeks after PCI, in accordance with current guidelines (Collet et al., 2021). Cardiologists determined the patients' eligibility for exercise intervention based on their clinical status and excluded those with severe cardiac abnormalities (e.g., heart failure, mitral stenosis, angina pectoris symptoms during the post‐PCI exercise test, atrial fibrillation, and neuropathy), that could potentially affect ECG morphology and HRV. Furthermore, the patients' population was categorized according to the change in exercise capacity after training into low, average, and high responders. Baseline characteristics in ACS groups were carefully examined with respect to LVEF, body composition, blood pressure, glucose metabolism, lipid profile, exercise capacity, medical history, and medication (Table 3). None of these clinical parameters differed between ACS groups. Therefore, clinical characteristics (e.g., blood pressure, lipid profile, glucose metabolism or severe left ventricle abnormalities at baseline) are unlikely to explain the differences in exercise training responses among the groups. Unfortunately, we do not have complete echocardiography data (e.g., atrial dimensions or any assessment of cardiac fibrosis) that could potentially affect PTF and P wave duration. The absence of comprehensive echocardiography data is the most significant limitation of this study. However, LVEF and the Sokolow–Lyon index did not differ between groups and were not associated with training response.

### 4.4 Heart rate variability and training response

The present study found no association between baseline HRV and training response in patients after ACS. The acute effect of the PCI procedure on HRV is unknown. However, temporal changes in HRV after acute myocardial infarction (from 1 week to 1 year) have shown a significant association with survival rate during a 5‐year follow‐up. Cardiac patients with reduced HRV after both time points (from 1 week to 1 year) are at the highest risk of cardiac death during the 5 years of follow‐up (Jokinen et al., 2003). Based on the present study, we do not have information on individual de novo HRV remodeling after PCI. It is likely that cardiovascular medication and the PCI procedure itself may have masked the role of HRV as a potential predictor of exercise training response.

## 5 STRENGTH AND LIMITATIONS

This is the first study investigating the association between individual exercise training responses and baseline ECG morphology and HRV following a 6‐month training program in patients after ACS. According to current guidelines (Collet et al., 2021), prescribing exercise training is mandatory for coronary patients after PCI. Therefore, using similar patients as a control group is ethically unacceptable. However, a healthy control group that did not undergo exercise training was included in the study. ANOVA for repeated measures with time × group interaction analysis showed a successful training response in patients after ACS at the group level compared to controls. The major limitation of the study is the lack of comprehensive echocardiographic examinations before and after exercise training. The number of female subjects in the patient population is also low, and a comprehensive evaluation of the effects of sex on PTF and training response requires further investigation. Finally, while a high correlation with repeated measurements of the same observer has been reported previously (Eranti et al., 2014), inter‐ and intraobserver variability for PTF measurements has not been established and is needed.

## 6 CONCLUSION

PTF analyzed from the standard ECG at resting condition after 1–3 weeks of discharge of patients with acute coronary syndrome predicts individual exercise training response assessed as exercise capacity. If PTF is observed on an ECG in a patient after a coronary event, they may have a reduced exercising training response and further diagnostics may be warranted to potentially improve the outcome of these patients. Of course, this observation requires confirmation in future studies.

## AUTHOR CONTRIBUTIONS

This study was conducted in 2017–2019 at the University of Oulu, Oulu, Finland. Conception and design of the work: Mikko Tulppo and Antti Kiviniemi. Acquisition analysis, and interpretation of the data for the work: Mikko Tulppo, Antti Kiviniemi, Elina Roiha, Juha Perkiömäki, Heikki Huikuri. Drafting the work and revising it critically for important intellectual content: Mikko Tulppo, Antti Kiviniemi, Elina Roiha, Juha Perkiömäki, Juhani Junttila, Heikki Huikuri. Final approval of the version to be published: Mikko Tulppo, Antti Kiviniemi, Elina Roiha, Juha Perkiömäki, Juhani Junttila, Heikki Huikuri. Agreement to be accountable for all aspects of the work: Mikko Tulppo, Antti Kiviniemi, Elina Roiha, Juha Perkiömäki, Juhani Junttila, Heikki Huikuri.

## FUNDING INFORMATION

Funding from EuroStars/Business Finland Grand number 3692/31/2015 and The Finnish Foundation for Cardiovascular Research, Helsinki, Finland (Project numbers 200190, 230117).

## CONFLICT OF INTEREST STATEMENT

The authors report no conflicts of interest.

## Data Availability

The data that support the findings of this article are not publicly available due to privacy and ethical concerns. They can be requested from the author at mikko.tulppo@oulu.fi.
